# Supplementary material for: Therapeutic efficacy of Chloroquine for the treatment of uncomplicated Plasmodium vivax infection in Shewa Robit, Northeast Ethiopia
Source: PLoS One. 2023 Jan 12;18(1):e0277362. doi: 10.1371/journal.pone.0277362 (PMC9836259; doi:10.1371/journal.pone.0277362)
Supplement: S1 File — (DOCX) [file pone.0277362.s002.docx]

**Patient screening form**

| 1 | Patient aged 6 months and over | Yes:□ | No:□ |
| --- | --- | --- | --- |
| 2 | The patient has severe malnutrition | Yes:□ | No:□ |
| 3. | The patient has mono-infection with *P.vivax* | Yes:□ | No:□ |
| 4. | Bodyweight 5 kg or more | Yes:□ | No: □ |
| 5. | Patient with fever or history of fever in the previous 24 hours | Yes: □ | No:□ |
| 6. | Non-pregnant or breast-feeding female | Yes:□ | No:□ |
| 7. | Residents living within 10 km radius of the health centre | Yes:□ | No:□ |
| 8. | 12. Evidence of concomitant febrile illness  If “YES”, indicate illness. If “NO”, leave blank. Pneumonia/RTI □ Measles□ Otitis Media □ UTI □ Gastroenteritis □ Other: □ | Yes:□ | No: □ |
| 9. | Evidence of severe malaria/danger signs  If “YES” indicates criteria. If “NO”, leave blank  □Unarousable coma (if after convulsion, > 30 min)  □Repeated convulsions (> 2 within 24 h)  □Recent convulsions (1-2 within 24 h)  □Altered consciousness (confusion, delirium, coma)  □Lethargy  □Unable to drink or breastfeed  □Vomiting everything  □Unable to stand/sit due to weakness  □Severe anaemia (Hb < 5.0 gm/dL)  □Respiratory distress (laboured breathing at rest)  □Jaundice (yellow colouring of eyes) | Yes:□ | No:□ |

**Enrollment form**

| Age…………….. | Gender/Male……Female……… | Weight……………… |
| --- | --- | --- |
| 1. Study  Number:---------- | 2. Number of tablets--------- | 3. Start Date ----------------- |
| 4. Patients Full name: | | |
| 5. Family head: | | |
| 6. Mother’s/Wife’s (if married) name: | | |
| 7. Caregiver’s name and relationship: | | |
| 8.Kebele/Street: | | |
| 9. Home parish: | | |
| 10. LC1/village: | | |
| 11. Home address and localizing features/Owners name/Direction: | | |
| Phone number (s) and the owner(s): | | |
| 12. Previous malaria attack: Yes _____No _____  13. Previous antimalarial intake: Yes ______No ______    If yes, CQ SP AL?  14. Hold Bed net: Yes ___No If yes, Bed net use Yes No ___­­­____  ______________________________________________________ | | |

**Case screening form**

| Case screening form | | |
| --- | --- | --- |
| Health centre name  Locality  District  Province | | Study number ------------------------  Patient screening number ------------------  Date of visit ---------------------------- |
| Demographic data | | |
| Age ----------- in a month------------------- in a year------------  Height ------------  Sex -----------  If the female is the patient pregnant? Yes ---------- No---------  If pregnant, provide the date of the last menstrual period -------------------------- | | |
| Pre-treatment temperature | | |
| History of fever in previous 24 hr? Yes ----------- No --------------  Temperature ------ ^0^C axillary---------- | | |
| Thick and thin blood smear for estimation of *p.vivax* parasite counts | | |
| Species: *P.falciparum*------- *P.vivax*----------  Were species other than *P.vivax* present? Yes --------- No ---------- (if yes, the patient is not eligible)  An approximate number of *P.vivax* asexual parasites:  Presence of 1-100 parasites / 3-6 white blood cells? Yes-------- No ----------( if no, the patient is not eligible)  Presence of *P. vivax* gametocytes? Yes ---------- No ----------  Has a blood sample for PCR been collected? Yes ------- No ---------  Haemoglobin ----------g/dl Haematocrit ----------------% | | |
| Urinary analysis (pregnancy test for female patients) | | |
| Result of pregnancy test: Positive ---------- Negative --------- (If positive, the patient is not eligible) | | |
| Does the patient meet any of the exclusion criteria? Yes -------- No -------- ( If yes, the patient is not eligible) | | |
| If Yes, please specify the reason for exclusion: | | |
| Patient informed consent and assent | | |
| Consent form signed Yes --------- No -------  Assent form signed Yes ---------- No ------- | Patient identity number -----------------  Date -------------------- | |

**Case Record Form**

PIN………… No. of Tablets ………………. Name…………………

| Follow up day | 0 | 1 | 2 | 3 | 7 | 14 | 21 | 28 | Extra day |
| --- | --- | --- | --- | --- | --- | --- | --- | --- | --- |
| Date |  |  |  |  |  |  |  |  |  |
| Successes of treatment ⁕ | 1  ------ | 2  ------ | 3  ----- |  |  |  |  |  |  |
| Axillary T^0^ |  |  |  |  |  |  |  |  |  |
| Parasite asexual |  |  |  |  |  |  |  |  |  |
| Gametocyte count |  |  |  |  |  |  |  |  |  |
| Haemoglobin |  |  |  |  |  |  |  |  |  |
| Adverse event ** |  |  |  |  |  |  |  |  |  |
| Concomitant treatment |  |  |  |  |  |  |  |  |  |
| Reason for withdrawal |  |  |  |  |  |  |  |  |  |
| Remarks |  |  |  |  |  |  |  |  |  |
| Completed by (initials0 |  |  |  |  |  |  |  |  |  |

⁕1. Observed by the health professional and successfully took medication

**1) Headache 2) Anorexia 3) Nausea 4) Vomiting 5) Abdominal pain 6) Diarrhea 7) Cough 8) Behavioral Change 9) Dizziness 11) Mouth ulcer 12) other, specify

**Laboratory Request Form**

| 1. **Client details**   Study participant ID ___________________ Study arm CQ ----------  Age---------- Sex Male -------- Female ----- |
| --- |
| 1. **Follow-up visit 3. Laboratory tests**   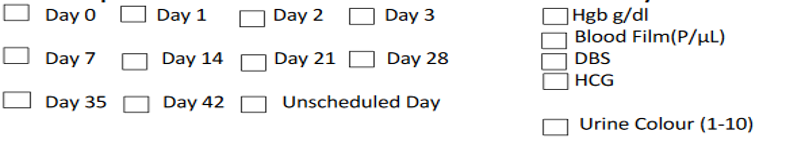 |
| 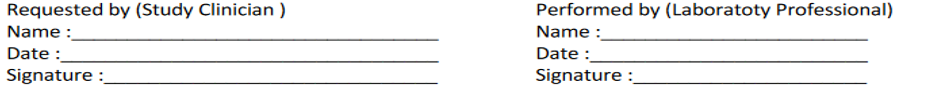 |

**Patient follow–up card**

**Patient Follow-up Card**

Patient Identification Number: ____________

Name-----------------------------------------------------

Scheduled visit day

| Day | 0 | 1 | 2 | 3 | 7 | 14 | 21 | 28 |
| --- | --- | --- | --- | --- | --- | --- | --- | --- |
| Appointment  date |  |  |  |  |  |  |  |  |

Note:
