# Supplementary material for: Therapeutic efficacy of Chloroquine for the treatment of uncomplicated Plasmodium vivax infection in Shewa Robit, Northeast Ethiopia
Source: PLoS One. 2023 Jan 12;18(1):e0277362. doi: 10.1371/journal.pone.0277362 (PMC9836259; doi:10.1371/journal.pone.0277362)
Supplement: Attachment — Submitted filename: renamed_058a5.pdf [file pone.0277362.s003.pdf]

Dear,

Congratulations on the study, it has great scientific relevance. Therefore, some questions need to be answered:

When analyzing your study, both the **abstract and the introduction** are contemplating the necessary information; The **methodology** needs to be further explored. It is still not entirely clear what the inclusion and exclusion criteria for the recruited patients were. In addition, the sample number seems small to me. Wouldn't it have a chance to increase? Another important factor is that no analysis of the biochemical parameters of the patients was performed, these data would give you significant answers to your findings. Why wasn't it done? The **results** are well described, but at the same time I noticed that the patients did not present itching. Typically, patients who use CQ have these reactions. Was it not identified or did these patients have and/or abandoned the segment? Furthermore, I suggest that in figures 3 and 4 you put the corresponding values in each highlight point for a better understanding for the reader; The **discussion** presented includes the necessary information. However, by addressing minor limitations you could emphasize that the process of genetic polymorphism could account for drug resistance. This is constantly being discussed in several scientific articles in different locations worldwide. It is also necessary for you to present more accurate information to justify your hypotheses and/or findings; The **conclusion** is ok. Also, what makes your work different?

I hope that this information has been essential for a better understanding of the study.

Thank you very much in advance.
